# Angiotensin-(1–7) as a Potential Therapeutic Strategy for Delayed Cerebral Ischemia in Subarachnoid Hemorrhage

**DOI:** 10.3389/fimmu.2022.841692

**Published:** 2022-03-09

**Authors:** Filippo Annoni, Federico Moro, Enrico Caruso, Tommaso Zoerle, Fabio Silvio Taccone, Elisa R. Zanier

**Affiliations:** ^1^Laboratory of Acute Brain Injury and Therapeutic Strategies, Department of Neuroscience, Mario Negri Institute for Pharmacological Research IRCCS, Milan, Italy; ^2^Department of Intensive Care, Erasme Hospital, Free University of Brussels, Anderlecht, Belgium; ^3^Neuroscience Intensive Care Unit, Department of Anesthesia and Critical Care, Fondazione IRCCS Ca’ Granda Ospedale Maggiore Policlinico, Milan, Italy; ^4^Department of Pathophysiology and Transplantation, University of Milan, Milan, Italy

**Keywords:** renin–angiotensin system (RAS), delayed cerebral ischemia (DCI), subarachnoid hemorrhage (SAH), anoxic injury, acute brain injury

## Abstract

Aneurysmal subarachnoid hemorrhage (SAH) is a substantial cause of mortality and morbidity worldwide. Moreover, survivors after the initial bleeding are often subject to secondary brain injuries and delayed cerebral ischemia, further increasing the risk of a poor outcome. In recent years, the renin–angiotensin system (RAS) has been proposed as a target pathway for therapeutic interventions after brain injury. The RAS is a complex system of biochemical reactions critical for several systemic functions, namely, inflammation, vascular tone, endothelial activation, water balance, fibrosis, and apoptosis. The RAS system is classically divided into a pro-inflammatory axis, mediated by angiotensin (Ang)-II and its specific receptor AT_1_R, and a counterbalancing system, presented in humans as Ang-(1–7) and its receptor, MasR. Experimental data suggest that upregulation of the Ang-(1–7)/MasR axis might be neuroprotective in numerous pathological conditions, namely, ischemic stroke, cognitive disorders, Parkinson’s disease, and depression. In the presence of SAH, Ang-(1–7)/MasR neuroprotective and modulating properties could help reduce brain damage by acting on neuroinflammation, and through direct vascular and anti-thrombotic effects. Here we review the role of RAS in brain ischemia, with specific focus on SAH and the therapeutic potential of Ang-(1–7).

## Introduction

Although aneurysmal subarachnoid hemorrhage (SAH) accounts for only 5% of all strokes, it is a substantial cause of premature death and disability, affecting 10 individuals per 100,000 every year, with a mortality rate of nearly 50% (1–3). Aneurysmal SAH affects younger adults than ischemic stroke and accounts for 27% of all stroke-related years of life lost before age 60 (4), and survivors commonly suffer cognitive and functional impairments (5). Thus SAH is a disease with an important personal and socio-economic impact, for which therapeutic strategies are urgently needed.

Patients surviving the early brain injury related to intracranial bleeding risk re-bleeding, hydrocephalus, seizures, intracranial hypertension, cardiac and pulmonary complications and delayed cerebral ischemia (DCI) (6). Early surgical or endovascular clipping/coiling of the aneurysm can significantly reduce the risk of re-bleeding. Despite preclinical and clinical research efforts (2, 7), strategies to prevent or limit DCI are limited. DCI is a major determinant of poor outcome, with an estimated 30% occurrence rate in SAH survivors (8) and its detection is still a challenge.

Clinical deterioration and/or a new cerebral ischemic lesion detected on cerebral computed tomography (CT-scan) or magnetic resonance imaging (MRI) are essential for the diagnosis of DCI (9). However, these approaches are of limited use in sedated or unconscious patients and offer only a few possible interventions, as cerebral ischemia is already present (10). Therefore additional diagnostic tools, namely, transcranial Doppler, electroencephalography, and cerebral CT-perfusion, have been proposed to optimize the diagnosis of DCI, although their potential is still debated (11).

One of the key determinants of DCI is cerebral vasospasm (CVS), defined as narrowing of the lumen of one or more of the major intracranial arteries (12). However, other mechanisms play a role. Blood extravasation after SAH leads to the exposure of the cerebral tissue to numerous intravascular components, triggering a local inflammatory response (13). This response is further aggravated by the accumulation of free radicals caused by the degradation of cellular components of the clot (14), ultimately generating a self-promoting detrimental loop (15). Microvascular dysfunction and cortical spreading depolarization can also occur in these patients and contribute to the DCI pathogenesis (6).

About two thirds of SAH patients develop CVS within two weeks of the aneurysmal rupture (16) and half of these will also develop DCI (6). These, patients often require intensive care for multimodal neuromonitoring and supportive care to minimize secondary cerebral injuries of systemic origin induced by fluctuations in blood pressure, arterial CO_2_ concentrations, hemoglobin levels, oxygen demand (i.e., core temperature, adequate sedation) and sodium levels (17).

Besides oral nimodipine, which has been associated with improved neurological outcome though with no significant reduction of CVS (18), treatments have aimed to treat large-vessel spasm (i.e., intravenous and intra-arterial vasodilators; balloon angioplasty), increase cerebral perfusion (i.e., controlled hypertension, milrinone, dobutamine), reduce systemic inflammation (i.e., statins, steroids) (11, 18–20), and maintain brain homeostasis (i.e., temperature control). None of these specifically target the multiple pathophysiological mechanisms involved in DCI.

### The Renin–Angiotensin System (RAS)

In recent years, the renin–angiotensin system has been proposed as a possible therapeutic target in different brain-related pathological conditions. In the so-called “classical RAS”, angiotensinogen is transformed by renin into angiotensin (Ang)-I, which is then cleaved into Ang-II by the angiotensin-converting enzyme (ACE); this than exerts its effects through the AT_1_ receptor (AT_1_R) or the AT_2_R subtypes, tuning water and salt homeostasis and modulating inflammation, fibrosis, apoptosis, and vascular tone (21, 22). AT_1_R stimulation induces vasoconstriction, cell proliferation, protein phosphorylation, sodium retention, fibrosis and oxidative stress, while AT_2_R activation counterbalances these effects (23). Although Ang-II binds with high affinity to both receptors, their expression widely differs throughout the body (24). The increase in Ang-II production results in a predominant AT_1_R-mediated effect, namely, vasoconstriction, as shown in the ATHOS trials (25).

**Figure 1:**
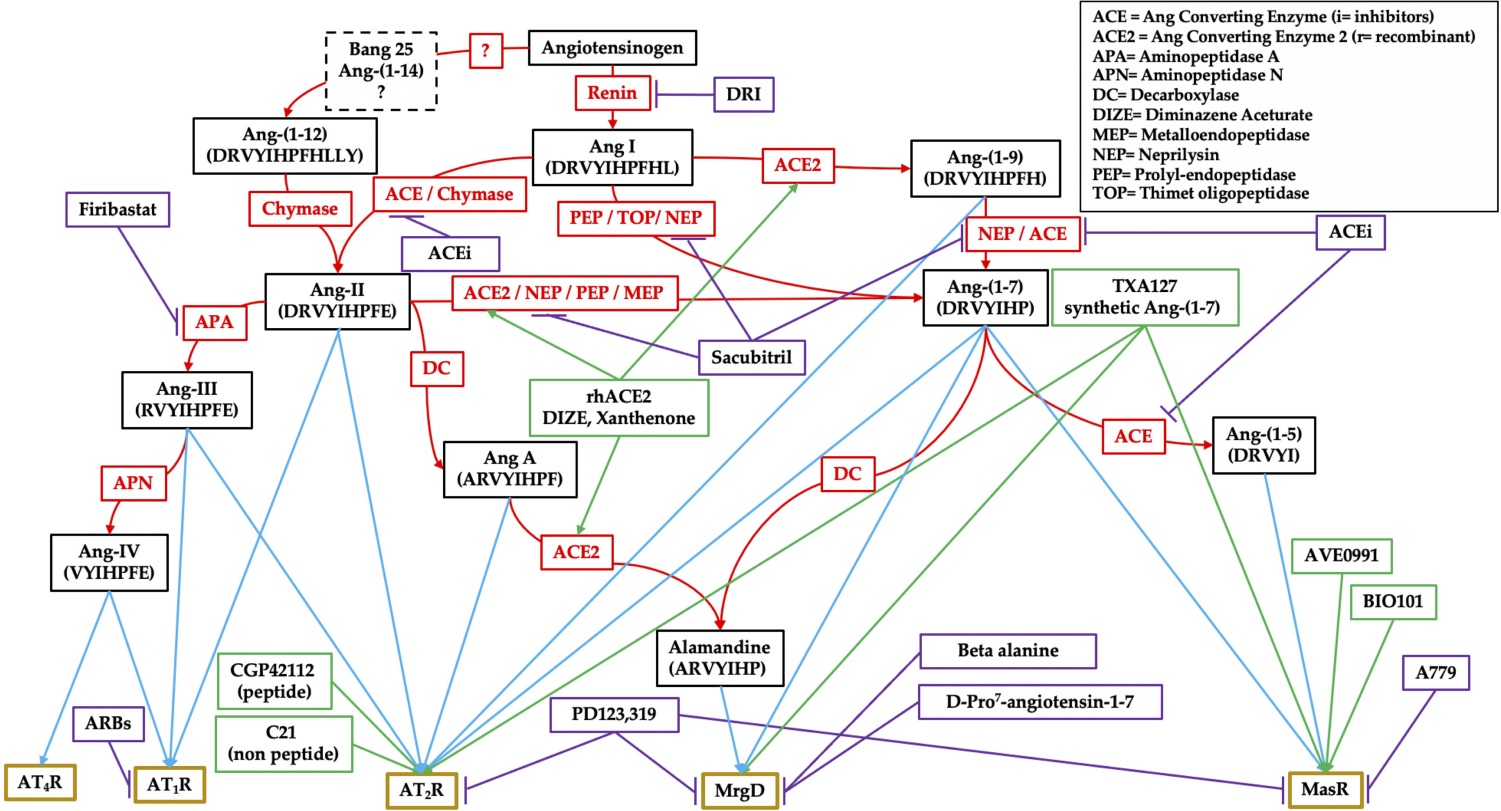
Schematic representation of the renin–angiotensin system (RAS). Black boxes are for the effectors, red ones for enzymes, purple ones for pharmacological inhibitors, green ones for pharmacological stimulators, and gold boxes for receptors.

Ang-II can also be transformed, by aminopeptidase A (APA), into Ang-III, which can stimulate the AT_1_R (26), and is then further processed by aminopeptidase N (APN) into Ang-IV, which has high affinity for the AT_4_R subtype (27). However, an alternative RAS pathway has been flanked and characterized as a counterbalancing system. This pathway has Ang-(1–7) as its main effector, derived either from Ang-II through the action of the monopeptidase ACE2, or through an intermediate transformation of Ang-I into Ang-(1–9) by ACE2 before its final conversion to Ang-(1–7) by ACE (**Figure 1****)**. By binding to its specific receptor MasR, Ang-(1–7) exerts a wide range of effects that counteract the pro-inflammatory, pro-apoptotic, pro-fibrotic and vasoconstrictive effects of the AT_1_R stimulation induced by Ang-II (28).

Ang-(1–7) can be further processed into two biologically active compounds, Ang-(1–5), also capable of binding to MasR, and alamandine, which binds to a specific MasR-related G-protein coupled receptor member D (MrgD), which is active in all the effects produced by this alternative RAS pathway (29, 30). This simplistic representation has led to the misconception of a “positive” RAS as opposed to a “negative” one. However, as suggested by its phylogenesis, RAS should be seen rather as a fine regulatory system, with high complexity, inter-human variability and widespread presence throughout the body (31). RAS is a complex network of interconnected tissue-specific and systemic RAS reactions that finely tune physiological functions.

In recent years several new angiotensins, receptors and enzymes have been discovered and characterized, and a numerous of compounds have been developed with the purpose of stimulating or blocking key components of both RAS pathways in many diseases. To combat hypertension and cardiac failure pharmacological RAS manipulations have aimed to inhibit the overstimulation of AT_1_R, with selective or unselective blockade (sartans or ACE inhibitors), through renin inhibition (aliskiren) or combined AT_1_R/neprilysin inhibition (sacubitril) (32). The antihypertensive drug sacubitril can block neprilysin, raising the concentration of natriuretic peptides and, in association with AT_1_R blockade, preventing the detrimental increase in Ang-II (33).

Given the complexity of RAS interconnections with other systems such as bradykinins, natriuretic peptides and prostaglandins, its manipulation has often produced differing results—as with omapatrilat (a neprilysin blocker combined with an ACE inhibitor), where the beneficial effects of blocking neprilysin are counterbalanced by the risk of angioedema due to the double blockade in the breakdown of bradykinin (34). However, no compound has been developed commercially to specifically target the alternative RAS by stimulating MasR, MrgD or AT_2_R-related pathways.

This approach has been proposed recently, in the context of the COVID-19 pandemic, as it appears that RAS may become unbalanced during the acute inflammatory response (35), and upregulation of the ACE2/Ang-(1–7)/MasR pathway could re-establish homeostasis within the RAS. Clinical trials evaluating the efficacy of MasR, AT2R agonists, the synthetic form of Ang-(1–7) and recombinant ACE2 in COVID-19, but no data are available about vasculature or cerebral tissues. A synthetic form of Ang-(1–7) has been given to women to treat pre-eclampsia, with vasculature-related endpoints—with some promising results (36).

Despite the large amount of evidence supporting the beneficial role of Ang-(1–7) in various conditions, particularly, cardiovascular disease, cancer, renal failure, and neurological disorders (37), problems related to the peptide’s short half-life and rapid plasmatic metabolism still limit its clinical applications (38).

Given the multifaceted nature of brain damage after SAH, an ideal therapeutic agent in this context should be capable of modulating inflammation, mitigating ischemic injury, and also providing favorable vascular and systemic effects, avoiding hypotension, which could further impair cerebral perfusion. With its favorable local and systemic modulatory effects on most SAH pathophysiological mechanisms, ACE2/Ang-(1–7)/MasR axis manipulation offers a possible new therapeutic target to prevent or mitigate DCI. This article reviews the evidence behind RAS manipulation in acute brain injury, with a focus on ischemia, neuroinflammation, the role of the alternative RAS, and the rationale for the potential use of Ang-(1–7) in the future to prevent DCI in SAH.

### Cerebral RAS

Since its first observation over half a century ago (39, 40), the presence of the RAS in the brain and its relationship with systemic physiological functions, such as blood pressure control and water–salt homeostasis, has been extensively investigated. Brain RAS has been implicated in the regulation of arterial blood pressure, body temperature, thirst and hydromineral balance, vasopressin release, adrenocorticotropin synthesis and memory (41, 42). All components of the classical and alternative RAS have been described in the brain (41), namely, MasR, Ang-(1–7), and Ang-(1–9) (43). In particular, renin and angiotensinogen are synthesized mainly by astrocytes, and a small percentage by neurons (44). The presence of AT_1_R and AT_2_R has been confirmed in dopaminergic neurons, astrocytes, and glial-cells (45–47), and ACE and ACE2 are expressed in many areas of the mammalian brain (48).

It is still not clear which molecule is the principal effector of the classical pathway in the brain. Ang-III, which is the product of Ang-II cleavage by APA, mediates several RAS pressor effects in the brain (49, 50). For this reason APA inhibitors are being investigated as novel antihypertensive drugs, targeting brain RAS and acting on vasopressin release and sympathetic tone (51). Ang-III is further processed by the APN into Ang-IV, whose receptor (AT_4_R) has a role in memory (52, 53). Lastly, Ang-(1–7) has also been linked to fine regulation of inflammation, oxidative stress, metabolic homeostasis, angiogenesis, and motor control and cognitive behavior in the brain (37).

### RAS in Brain Ischemia

In ischemic stroke, therapeutic manipulation of brain RAS has been investigated to control blood pressure (54) and blockade of the classical RAS with ACE inhibitors and selective AT_1_R inhibitors (ARBs) has proven more effective than beta-blockers for secondary prevention of stroke (55). The relationship between RAS and stroke has been recently summarized (56, 57). Briefly, AT_1_R-deficient mice have a larger penumbra area and a smaller area of energy failure than wild-type littermates after middle cerebral artery occlusion (MCAO) (58). It is still not clear whether ARBs have a neuroprotective effect mediated by the decrease in AT_1_R activation signaling pathways or the AT_2_R indirect stimulation, but rats immunized with Ang-II peptide vaccine prior to MCAO had less neurodegeneration through suppression of the brain RAS and reduction of oxidative stress (59). In addition, the infarct was smaller, and neurological performance was better in animals given an AT_2_R agonist (compound 21 or CGP42112) after MCAO (60, 61).

The alternative ACE2/Ang-(1–7)/MasR pathway is also directly involved in the pathophysiology of ischemic stroke, and the expression of both ACE2 and MasR is upregulated after acute stroke in rats. These animals have higher regional and circulating levels of Ang-(1–7), suggesting that this axis could play a key role in the response to brain ischemia (62). There is also experimental evidence of direct neuroprotective effects of Ang-(1–7). Neurological outcome and infarct size improved when Ang-(1–7) was injected in the brain ventriculi of rodents prior to endothelin-1 mediated MCAO (63). Intraventricular Ang-(1–7) also reduced infarct size and inflammation (64) after permanent MCAO in rodents. The concomitant injection of the MasR antagonist A-779 led to similar findings, corroborating a protective action of Ang-(1–7). Interestingly, there were still beneficial effects when Ang-(1–7) was given orally at various times after reperfusion (65). Furthermore, in spontaneously hypertensive rats, prone to hemorrhagic stroke, centrally administered Ang-(1–7) increased the lifespan and improved neurological performance (66). Recently, intranasal administration of AVE 0991, a MasR agonist in an experimental model of SAH was associated with improved neurobehavioral function and a reduction in oxidative stress and neuronal apoptosis (67). These results partially contrast with previous findings in an *in vivo* model of ischemic stroke, where centrally injected AVE 0991 did not improve functional or histological endpoints, but did protect ischemic neurons *in vitro* (68).

Targeting ACE2 might also be an important therapeutic option for ischemic stroke (69). Mice overexpressing ACE2 in neurons are protected from ischemic injury through regulation of the NADPH oxidase and endothelial nitric oxide synthase pathways and show a reduction of reactive oxygen species (ROS) after ischemic stroke (70).

The ACE2 increase by central or systemic diminazene aceturate (DIZE) after ischemic stroke improved neurological function and reduced the infarct volume (71). Thus, data suggest that RAS mediates early and delayed consequences after experimental stroke (72, 73) and RAS genetic polymorphisms are thought to be involved in cerebral aneurysm formation and rupture (74), whether these observations can be translated to SAH and/or DCI remains to be shown.

### RAS and Neuroinflammation

Even though systemic RAS does not act directly on the central nervous system, tissue-specific RAS is found in the brain and is implicated in several physiological and pathological processes. Local brain RAS may have pro-inflammatory action and pro-fibrotic effects through the activation of the ACE/Ang-II/AT_1_ axis but also mediates anti-inflammatory effects through the activation of the alternative ACE2/Ang-(1–7)/MasR or AT_2_R (37).

Microglia are resident brain immune cells responsible for the elimination of microbes, dead cells, protein aggregates, and other dangerous substances or debris, with a key role in orchestrating neuroinflammatory changes (75). Importantly, AT_1_R and AT_2_R receptors are present in activated microglia, and RAS stimulation elicits direct activation of microglia cells *in vitro* and release of pro-inflammatory cytokines through a NF-kB mediated mechanism (76). RAS-mediated microglial inflammation is mainly associated with Nox-derived ROS that can also interfere on intracellular signaling pathways involved in microglial activation, stimulating the release of proinflammatory signals (77).

Together with microglia, astrocytes are important sources of pro- and anti-inflammatory cytokines, they also sustain blood–brain barrier, provide metabolic support to neurons, and maintain synaptic homeostasis (78, 79). Ang-II regulates astroglial functions by inducing astrocyte proliferation and activating intracellular signaling pathways linked to inflammatory status, cellular growth, proliferation and increases in blood–brain barrier permeability, thus favoring the migration of peripheral immune cells into brain tissue (80).

**Figure 2:**
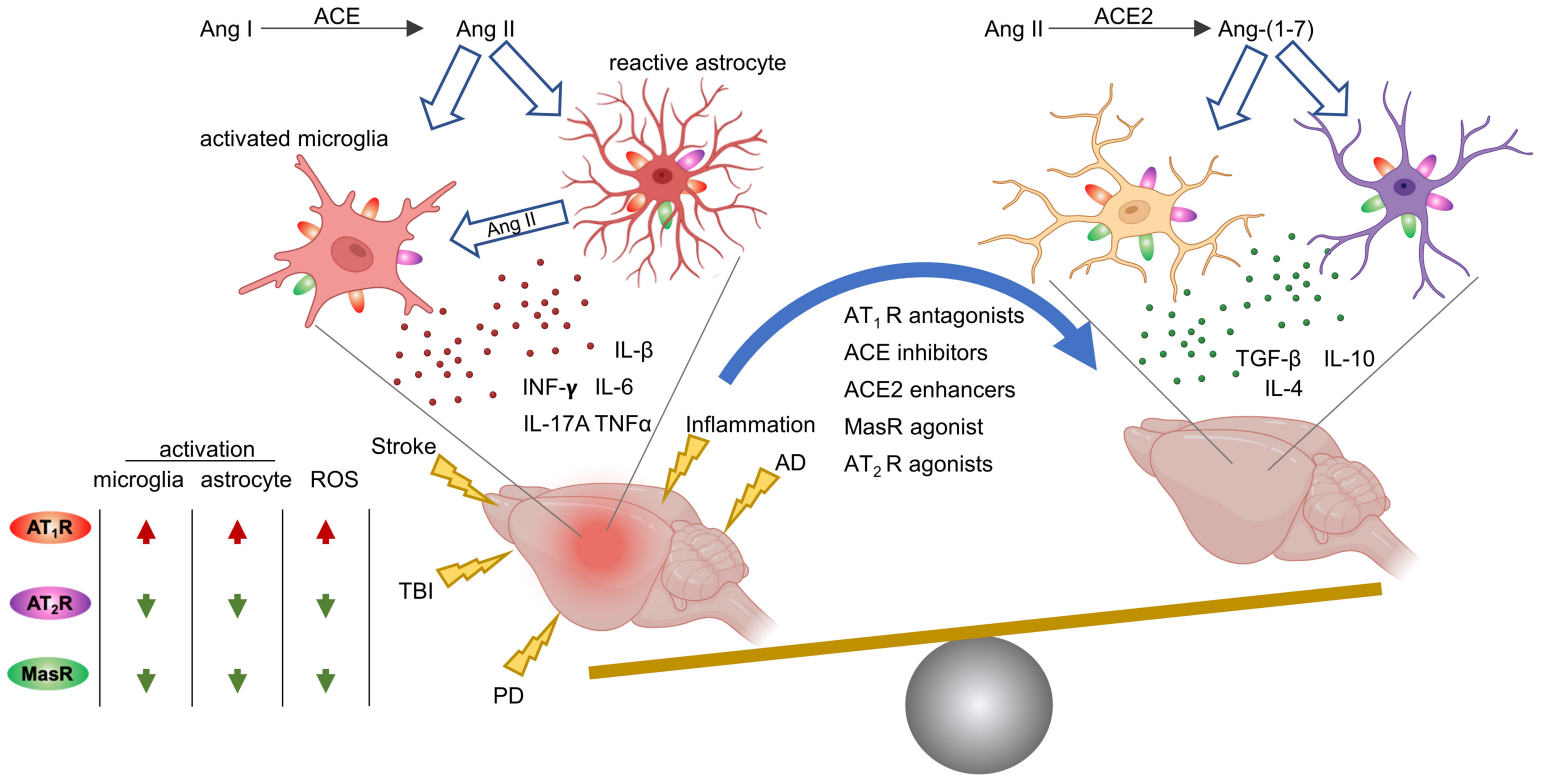
RAS modulation through AT_1_R, AT_2_R, MasR counteract neuroinflammation across several brain pathological brain conditions. PD, Parkinson’s disease; AD, Alzheimer’s disease; TBI, traumatic brain injury; ACE, angiotensin-converting enzyme; Ang, angiotensin; ROS, reactive oxygen species. Image created with BioRender.com.

There is growing evidence that RAS can be effectively targeted to reduce neuroinflammation in several brain pathologies, namely, Alzheimer (81–86), Parkinson (87–89), ischemic stroke (90, 91), traumatic brain injury (TBI) (92, 93), hypertension (94, 95) and inflammation (96) (**Figure 2**). Reduced astro- and micro-gliosis has been reported after blockade of the ACE/Ang-II/AT_1_R pathway, leading to a reduction in cytokine production and a better functional outcome (81, 83, 86, 90, 93, 96, 97).

Recent data indicate that vaccination against Ang-II inhibited astrocytic and microglial activation by stimulating basic fibroblast growth factor 2 (FGF2) signaling, and improved cognitive outcome in rats with vascular dementia (59). Neuromodulatory effects mediated by microglia and astrocytes have been reported with ARBs (82, 85, 86, 95, 98–100) and ACE inhibitors (ACEi) (82). Also, AT_1_R knock down by viral vector, reduced inflammatory mediators and glial activation in hypertensive rats (101). A shift of microglial polarization toward a more protective phenotype has also been observed after either direct stimulation of the AT_2_R receptor by Compound 21, a potent AT_2_R agonist (102), or AVE 0991 MasR agonist (103, 104) or ACE2 stimulation (103).

Interestingly, sexual dimorphisms seem implicated in RAS activity on neuroinflammation. Estradiol regulates the expression of AT_1_R and ACE activity in several peripheral tissues (105) and mediates dopaminergic cell damage in Parkinson’s disease (106). In experimental models of Alzheimer’s disease inhibition of the ACE/Ang-II/AT_1_R axis by ARBs (98) or ACEi (82, 84) had higher anti-inflammatory effects in the brain of female mice. In line with these observations, the stimulation of estrogen receptor β counteracts the negative effect of Ang-II on microglial polarization (77), highlighting a potential link between low estrogen levels and Ang-II mediated neuroinflammation in microglia. Moreover, positive interactions between the level of estrogen and the expression of AT_2_R (107, 108) and Ang-(1–7)/MasR (108–111) have been reported at both peripheral and central levels. Female sex hormones upregulate brain ACE2/Ang-(1–7), with a protective role against experimental hypertension in mice by increasing estrogen receptor α and lowering Nox expression in the brain (112, 113). This effect is reversed after either natural or surgical menopause (112). Importantly, estrogen deficiency, as in the perimenstrual phase and menopause, has a role in aneurysm formation and rupture (114), with a higher incidence of SAH in women after the age of 40 (115).

Neuroinflammation has been proposed to contribute to the DCI and poor outcome in SAH patients (15). RAS modulation either by inhibiting ACE/Ang-II/AT_1_R or stimulating the ACE2/Ang-(1–7)/MasR or AT_2_R axis has shown anti-neuroinflammatory effects in several brain pathologies (**Figure 2**) but *in vivo* studies exploring the possibility of acting on RAS to improve SAH outcome are lacking and are needed in the future.

### Ang-(1–7) to Act on Neuroinflammation

The alternative ACE2/Ang-(1–7)/Mas axis exerts effects that appear to be opposite to those of Ang II (116). Like the classical RAS, the receptors of the alternative RAS too are present in microglia and astrocytes (46, 117–121). ACE2/Ang-(1–7)/MasR axis activation either by stimulating ACE2 (103), MasR agonist (104, 122) or with calcitriol (vitamin D) (94) shifts microglial polarization toward a less toxic phenotype. Ang-(1–7) itself can exert direct effects on microglial cells by reducing their activation and the release of pro-inflammatory cytokines, namely, interleukin-1β (IL-1β) and tumor-necrosis factor α (TNF-α), while increasing the anti-inflammatory cytokine interleukin-10 (123).

The option to employ Ang-(1–7) to reduce neuroinflammation has been recently explored in the experimental setting in brain pathologies (**Table 1**). The effect of Ang-(1–7) on microglial cells is mediated by inhibition of inducible nitric oxide synthase (iNOS) (120) and of the NF-kB pathway (64). Ang-(1–7) also has a positive effect on astrocytes through the regulation of MAP kinase signaling, namely, downstream mediators such as PKCα and MEK (134). Additionally, through inhibition of the MAPK/Nox signaling pathway and by acting on the inflammatory cascade HMGB-1/RAGE/NF-κB/TNF-α, Ang-(1–7) prevented neuronal damage in an experimental model of Parkinson’s disease (125). Ang-(1–7) given subcutaneously 6 h after experimental TBI showed promising anti-inflammatory and neuroprotective properties, with a reduction of astrogliosis and microgliosis, increased neuronal and capillary density, and better cognitive performance one month after TBI (129).

**Table 1:** *In vivo* studies showing the anti-inflammatory action of Ang-(1–7) in the brain.

Reference	Pathology	Species	Treatment	Neuroinflammation	Functional outcome	Other findings
Hoyer‐Kimura et al. (124)	Cognitive impairment	mouse	glycosylated Ang-(1–7) (PNA5), 50–500 μg/kg subcutaneously injected for 24 d	↓ pro-inflammatory cytokine (TNF-α) ↑cytokines (IL-1α, IL-2, IL-5, IL-13, IL-17, IL-10)	↑ memory	↓ NfL (both with Ang-(1–7) and PNA5)
Rabie et al. (125)	Parkinson’s disease	rat	Ang-(1–7), 240 pg daily injected into the striatum for 1 w	↓ pro-inflammatory markers (RAGE and HMGB-1, NF-κB, p65 TNF-α, PARP-1)	↑ motor performance	rescue of dopaminergic neurons
Arroja et al. (126)	Stroke	rat	Ang-(1–7), 1 nmol/h intracerebroventricular infusion with osmotic pump for 6 w	no effect		↓ tissue damage ↑ BBB damage ↑Nox1
Cao et al. (127)	Alzheimer’s disease	mouse	Ang-(1–7), 400 ng/kg/min, with osmotic minipump for 4 w	↓ microgliosis (CD68, IBA1)	↑ memory	↓ amyloid deposits ↑ neuronal count
Hay et al. (128)	Cognitive impairment	rat	glycosylated Ang-(1–7) (PNA5), 1.0 mg/kg subcutaneously for 21 d	↓ microglial activation (IBA1) ↓ pro-inflammatory cytokines (TNF-α, IL-7) ↑anti-inflammatory cytokine (IL 10)	↑ memory	PNA5: ↑ blood-brain permeability ↑ stability/bioavailability ↓ ROS
Janatpour et al. (129)	TBI	mouse	Ang-(1–7), 1 mg/kg s.c. by osmotic pumps 1 or 6 h post-injury, until 3 or 29 d.	↓ astrogliosis (GFAP) ↓ microgliosis (IBA1)	↑ learning and memory ↑ motor	↓ lesion volume ↓ neuronal death ↓ vessel density
Regenhardt et al. (66)	Stroke	rat	Ang-(1–7), 100 pg intracerebroventricular infusion with osmotic pump for 6 w	↓ microgliosis (IBA1) ↓ pro-inflammatory cytokines (IL-1α, IL-6)	↑ lethargy	↑ survival ↓ brain hemorrhages
Regenhardt et al. (120)	Stroke	rat	Ang-(1–7), 1.1 nM; 0.5 μl/h in the brain by osmotic pumps	↓ microgliosis (IBA1)		
Rabie et al. (130)	Parkinson’s disease	rat	Ang-(1–7), 240 pg daily injected into the striatum for 1 w	↓ pro-inflammatory markers (p-MAPK p38/NF-κB p65)	↑ motor performance	
Hay et al. (131)	Heart failure	mouse	Ang-(1–7), 500 pg/kg/h s.c. by osmotic pump for 4 w	↑ neuroprotection markers (CXCL12, CXCL13,G-CSF,CCL2,IL-16,IP-10,sICAM and IL-1ra)	↑ memory	
Goldstein et al. (132)	Brain damage by Shiga toxin	rat	Ang-(1–7), 200 pg daily injected into the hypothalamic area for 8 d	↓ microglial cell number ↓ astrocytic damage		↓ neuronal damage ↓ demyelination
Bihl et al. (133)	Stroke	mouse	Ang-(1–7) (240 pg/kg/h) minipump infusion	↓ pro-inflammatory markers (TNF-α, MCP-1, IL-8, NF-κB)	↓ sensorimotor deficits	↑ vascular remodeling ↓ hemorrhage volume

It has been recently proposed that the kinin system, RAS and complement system are closely interconnected and have a major role in regulating vascular tone and inflammation (135). Ang-(1–7), by AT_2_R-mediated signaling, can counter-regulate blood pressure elevation by stimulating bradykinin production (136) and boosting the bradykinin–NO–cGMP pathway (135). In pathological conditions, particularly SAH (137), over-activation of the complement system induces leukocyte recruitment and extravasation increasing vascular permeability that can lead to tissue edema (135). Thus, Ang-(1–7) given after SAH could restore the balance in vascular permeability (37), mitigating pro-inflammatory mechanisms in brain tissue and blood vessels.

Among pharmacological treatments targeting brain RAS, Ang-(1–7), by stimulating MasR and AT_2_R, can blunt brain and vascular inflammation and at the same time improve functional outcomes in pathological conditions. Importantly, direct stimulation of one of the MasR (one of the targets of Ang-(1–7)) reduced oxidative stress and neuronal apoptosis in experimental SAH (67). Nevertheless, since Ang-(1–7) might also activate AT_2_R in tissue there is the possibility that Ang-(1–7) might counteract central and vascular inflammatory processes after SAH.

### Potential Beneficial Effect of Ang-(1–7) in SAH

It has recently been proposed that inflammation and oxidative stress may be a common ground for most of the causes of DCI (6, 15). After SAH blood components trigger an inflammatory response in the brain (13), that is further aggravated by the production of free radicals caused by the degradation of red blood cells from the clot (14); this builds up a self-promoting detrimental loop where neuroinflammation causes oxidative stress and oxidative stress aggravates neuroinflammation. As noted above Ang-(1–7) has the potential to block this detrimental loop triggered by SAH, modulating micro- and astro-glial function.

#### Direct Vascular Activity

**Figure 3:**
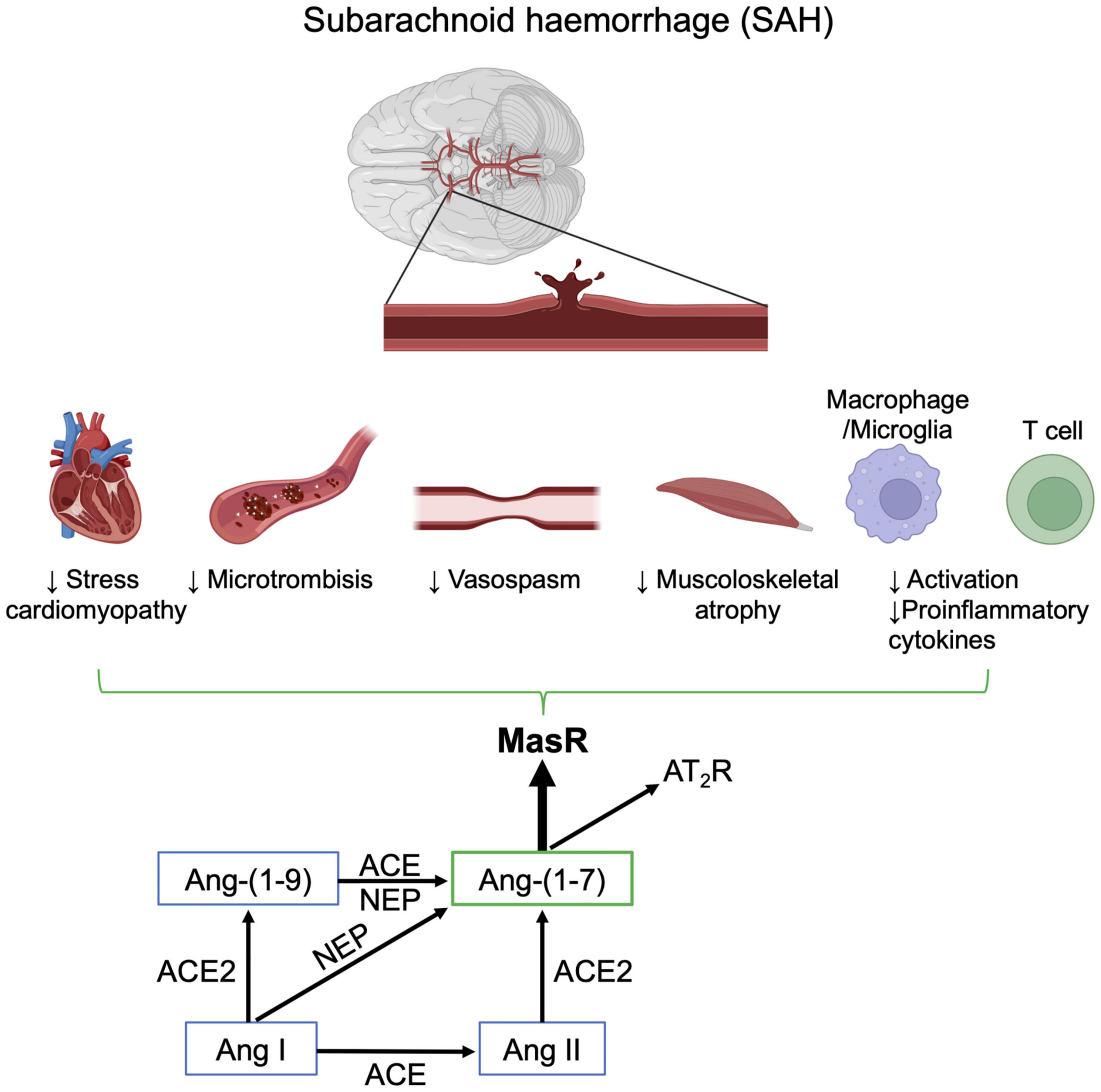
Schematic representation of the positive effects of Ang-(1–7) after subarachnoid hemorrhage (SAH). Image created with BioRender.com.

Besides its action on neuroinflammation, Ang-(1–7) has other potential beneficial effects in SAH (**Figure 3**). The vasodilating properties have been described in experimental models (138, 139). In healthy rats, Ang-(1–7) infusion modifies blood flow distribution, increases brain perfusion and vascular conductance, reduces vascular resistance in the brain and increases the cardiac index (140, 141). In situations of unbalanced RAS, such as in diabetic rats, Ang-(1–7) treatment restores carotid blood flow and lowers carotid resistance (142). In Ang-II induced hypertensive mice given intracerebral injections of elastance to increase the incidence of aneurysm, animals treated with Ang-(1–7) had a smaller proportion of ruptured intracranial aneurysms and lower mortality than controls through the MasR dependent pathway (143). The regional impact of Ang-(1–7) in humans has not been clarified yet, but it contrasted Ang-II-induced vasoconstriction in human artery fragments from patients treated for coronary revascularization. Interestingly, this action seems independent from MasR activation, AT_2_R or endothelium, and involves a direct effect on vascular smooth muscle cells (6). The effects of Ang-(1–7) on vasorelaxant compounds such as NO and prostacyclin have also been linked to the anti-thrombotic action (144–146). The relationship between Ang-(1–7) circulating levels and vascular effects is complex and is affected by comorbidities and concurrent therapies, such as ACEi (147–149).

#### Anti-Thrombotic Properties

Activation of the coagulation cascade and platelet activation are linked with DCI in SAH patients (150). The presence of microthrombi has been confirmed in two post-mortem studies (151, 152), but the clinical relevance of treatments to reduce thrombus formation is still not clear. Randomized controlled trials so far have found no benefit when aspirin was given to prevent platelet aggregation (153), or have given uncertain responses regarding the use of enoxaparin (154, 155), and guidelines are still based on low-quality evidence (11). In animals the Ang-(1–7)/MasR axis exerts significant antithrombotic effects after both acute and chronic administration (146) and the effect may be mediated by action on NO and prostacyclin release in platelets (144, 145) (**Figure 3**).

#### Musculoskeletal Atrophy

SAH patients requiring ICU care are often hospitalized for prolonged periods and suffer to a progressive decrease of muscle mass, with loss of strength and worsening outcome (156, 157). ICU-acquired weakness has been related to later hospital discharge, prolonged mechanical ventilation, and increased mortality (158). RAS has been proposed as a therapeutic target in musculoskeletal diseases, and while the classical RAS has been repeatedly linked with muscle fibrosis (159), Ang-(1–7) exerts a protective role in muscular dystrophy (160). Moreover, Ang-(1–7) prevented Ang-II-related catabolism (161) and muscle fiber shrinkage in an animal model of muscle atrophy due to immobilization (162) (**Figure 3**).

#### Stress-Induced Cardiomyopathy

Supranormal blood pressure thresholds (i.e., euvolemic hypertension) are often targeted in severe SAH patients after treatment of an aneurysm, to prevent regional hypoperfusion. Takotsubo syndrome (TTS), or stress-induced cardiomyopathy, is a life-threatening condition associated with SAH, due to increased endogenous and/or iatrogenic catecholamine load (163). In a propensity matched cohort, RAS manipulation using ACEi and ARBs, but not beta blockers, gave survival gain at one year and lower recurrence of the TTS (164). Ang-(1–7) levels were lower than to controls in an animal model of TTS by vagal electrical stimulation (165). The heart might nevertheless benefit from a higher systemic level of Ang-(1–7), as its levels have been associated with less heart failure after myocardial infarction (166), less myocardial swelling (167), and antiarrhythmic effects (168) (**Figure 3**). Ang-(1–7) infusion as a treatment for TTS still needs to be explored.

#### Adaptative Immune Cell Modulation

Mas signaling affects macrophage polarization, migration, and macrophage-mediated T-cell activation, all regulated by the alternative Ang-(1–7)/MasR axis (169). At the macrophage level, the lack of Ang-(1–7)-mediated inhibition on MasR results in enhanced T-cell proliferation *in vitro* co-culture experiments (170–172). In preclinical studies, activating AT_1_R receptors in T lymphocytes and myeloid cells blunts the polarization of these cells toward pro-inflammatory phenotypes (169) (**Figure 3**).

#### Pharmacological Considerations

Ang-(1–7) has a short half-life of ~0.5 h after subcutaneous injection, but it is promptly available and reaches its peak plasma concentration at ~1 h (173). Its bioavailability is even shortened when injected intravenously, as the peptide is rapidly degraded by circulating enzymes, namely, ACE, aminopeptidase A, and DDP3; this makes the development of a commercially distributed drug particularly challenging. In critically ill patients continuous intravenous infusion of Ang-(1–7) would be the safest administration route, achieving a tailored plasmatic increase and allowing for prompt discontinuation in case of hemodynamic instability. Clinically useful data are expected from ongoing clinical trials in severe COVID-19 patients (NCT04332666; NCT04570501; NCT04633772).

To overcome the unfavorable PK/PD profile of the compound, several stabilized forms are currently under investigation, namely, cyclic Ang-(1–7), cyclodextrins-included or bioencapsulated Ang-(1-7), modified amino acids and a new peptide Ang-1–6-O-Ser-Glc-NH2 (PNA5), which offer better brain-penetrating properties than Ang-(1–7). PNA5 given subcutaneously for 24 days reduced the expression of pro-inflammatory cytokines, cognitive impairment, and the plasma level of the axonal damage marker neurofilament light after myocardial infarction (124, 128).

Other pharmacological strategies to boost the alternative RAS include MasR agonists (AVE0991 and BIO101), AT_2_R agonist (Compound 21), and recombinant ACE2 (rhACE2). Detailed analysis of the characteristics of these treatments is beyond the scope of this review, but it is likely that these strategies will not be biologically equivalent. On the one hand rhACE2 could increase the generation of Ang-(1–7), but on the other it might also lower the overall plasmatic levels of Ang-II, as shown in patients with acute respiratory distress syndrome (174). Their impact after brain injury in patients has not been studied. ACE2 activators, namely, xanthenone and the antiparasitic drug DIZE have a more favorable PK/PD profile than to Ang-(1–7). DIZE appears to stimulate the alternative pathway of the RAS, with favorable cardiovascular, renal, and immune effects (175) but clinical evidence is limited on their effects in the brain. DIZE has been used with off-label in patients, with no toxicity reported (176). However, animal studies show potential drug-related brain toxicity, which appears to be species-dependent (177).

Selective MasR agonism might induce similar biological responses compared to Ang-(1–7), but without the concomitant generation of the derivates of the peptide such as alamandine and Ang-(1–5)—both biologically active. Selective AT_2_R agonism will not trigger MasR and MrgD cascades, thus only marginally affecting the alternative RAS.

## Conclusions

RAS components have numerous effects in the brain, and experimental evidence indicates that inhibiting the ACE/Ang-II/AT_1_R axis and stimulating the ACE2/Ang-(1-7)/MasR axis have positive effects in ischemic brain injury, particularly stroke. RAS might be important role in SAH and DCI, mainly increasing AT_1_R mediated oxidative stress and neuroinflammation and by modulating vascular changes that can promote CVS. To our knowledge, the activation of the alternative RAS has not been studied as a strategy to prevent DCI in SAH and only few studies have explored the potential beneficial effects of Ang-(1–7) after SAH, mainly focusing on its vascular effects. Further studies should test the pleiotropic activity of Ang-(1–7) and its potential to counteract Ang-II/AT_1_R activation to combat DCI.

## Author Contributions

FA, FM, and ERZ conceived the study. FA and FM contributed equally to writing the first draft of the manuscript. All authors listed have made a substantial, direct, and intellectual contribution to the work and approved it for publication.

## Funding

Partially supported by the Fondazione Cariplo (2019-1632). FA was supported with the mobility fund of the Fonds de Recherche Scientifique (FRS-FNRS).

## Conflict of Interest

The authors declare that the research was conducted in the absence of any commercial or financial relationships that could be construed as a potential conflict of interest.

## Publisher’s Note

All claims expressed in this article are solely those of the authors and do not necessarily represent those of their affiliated organizations, or those of the publisher, the editors and the reviewers. Any product that may be evaluated in this article, or claim that may be made by its manufacturer, is not guaranteed or endorsed by the publisher.
